# The epistemology of Deep Brain Stimulation and neuronal pathophysiology

**DOI:** 10.3389/fnint.2012.00078

**Published:** 2012-09-20

**Authors:** Erwin B. Montgomery

**Affiliations:** Department of Neurology, University of Alabama at BirminghamUSA

**Keywords:** Deep Brain Stimulation, epistemology, pathophysiology, mechanisms of action

## Abstract

Deep Brain Stimulation (DBS) is a remarkable therapy succeeding where all manner of pharmacological manipulations and brain transplants fail. The success of DBS has resurrected the relevance of electrophysiology and dynamics on the order of milliseconds. Despite the remarkable effects of DBS, its mechanisms of action are largely unknown. There has been an expanding catalogue of various neuronal and neural responses to DBS or DBS-like stimulation but no clear conceptual encompassing explanatory scheme has emerged despite the technological prowess and intellectual sophistication of the scientists involved. Something is amiss. If the scientific observations are sound, then why has there not been more progress? The alternative is that it may be the hypotheses that frame the questions are at fault as well as the methods of inference (logic) used to validate the hypotheses. An analysis of the past and current notions of the DBS mechanisms of action is the subject in order to identify the presuppositions (premises) and logical fallacies that may be at fault. The hope is that these problems will be avoided in the future so the DBS can realize its full potential quickly. In this regard, the discussion of the methods of inference and presuppositions that underlie many current notions is no different then a critique of experimental methods common in scientific discussions and consequently, examinations of the epistemology and logic are appropriate. This analysis is in keeping with the growing appreciation among scientists and philosophers of science, the scientific observations (data) to not “speak for themselves” nor is the scientific method self-evidently true and that consideration of the underlying inferential methods is necessary.

## The significance of deep brain stimulation (DBS)

The effectiveness and safety of Deep Brain Stimulation (DBS) for an expanding array of neurological and psychiatric disorders has been remarkable on a number of different levels. It is the most effective therapy for an expanding array of neurological and psychiatric disorders where DBS succeeds where all manner of medications, biologics, and gene therapies do not provide comparable benefit. In many ways, DBS harkens back to what may now be a lost notion, that the brain may have properties uniquely instantiated in the electrical activities distinct from those of neurotransmitters. If so, then physiology is not synonymous with neurotransmitter action, the contrary notion could reasonably argued has dominating much thinking in physiology and pathophysiology (Valenstein, 2005).

While many tens of thousands of patients have had dramatic improvements in the quality of their lives through DBS, a fair question is what has been the nature of that success. The enormous clinical benefits do not mean that there have been comparable advances in understanding the therapeutic mechanisms of action of DBS. In other words, it was not the case of reasoning as follows: DBS acts via mechanism A and it is known that disease X causes a change Y in structure Z that is amendable to mechanism A and consequently, DBS of structure Z should be effective in disease X. One merely has to look at the clinical targets of DBS to realize that they follow from previous targets of surgical ablations, with only rare exceptions. One exception has been DBS of the subgenu cingulum based on changes in neurometabolic imaging demonstrating increased metabolism in specific brain regions in patients refractory to standard therapies (Mayberg et al., 2005). However, even this development was based on what is now probably a false presupposition that high frequency stimulation inhibits or suppresses local neuronal activity (Montgomery and Gale, 2008). The other exception is DBS of the intralaminar nuclei of the thalamus for minimally conscious states (Schiff et al., 2007).

The concern noted above is not at all to deny the remarkable, indeed revolutionary contributions of DBS nor its potential to continue to make remarkable contributions. Nor does the concern deny the remarkable number of studies on DBS mechanisms ranging from recordings in reduced “slice” preparations to neurometabolic studies. However, the claim made here is that this very large cataloging of observations has not resulted in any clear understanding. Can anyone say which of the often conflicting observations is THE DBS mechanism?

## Issues of concern

The paucity of understanding DBS mechanisms is despite nearly a quarter of a century of renewed interest in brain stimulation for neurological disorders. Any scientist has to be concerned. The lack of progress cannot be merely attributed to the lack of research support. The reason, it is claimed here, lies with errors in much past and present research, which unless realized, will plague future research. The fact is the past and current experiments were technically sound, observationally true, and conducted by very talented and intelligent investigators. Yet, an understanding depends on how inferences are made from the data. Thus, the central claims of this discussion to be demonstrated is that many of the inferences that have and continue to be drawn are probably false as they are based on faulty presuppositions and logical errors. Just as scientists accept the appropriateness of discussions of experimental methods, it is hoped that the same appreciation will be extended to this discussion of inferential methods.

The purpose here is not to review all of the literature related to possible DBS mechanisms, which would be far beyond the scope of this article. Rather, the primary purpose is to review the epistemic nature of the inferences or claims made about the DBS mechanisms. Only examples from the literature that demonstrate or illuminate the epistemic issues are cited. As will be seen, there are at least two aspects to be discussed. First are the effects of logical fallacies on the progress in research on DBS mechanisms. The fundamental paradox is that the logical fallacies at first appear very reasonable, indeed central to the scientific method. As well be shown, these paradoxes are important in hypothesis generation but are counterproductive when confused for rules of evidence, as has been the case in DBS research.

The second issue relates to the question of “theory-free” observations. As will be demonstrated, what constitutes relevant experimental designs, observations and inferences draw heavily on presuppose theories of pathophysiology, for example the Globus Pallidus Interna (GPi) Rate theory that posits overactivity of the GPi as causal to parkinsonism. Again, the purpose here is not to extensively review the various theories of pathophysiology that have informed, or misinformed, DBS research. Rather, only selected aspects of these theories that illustrate the effects of presuppositions on DBS research are cited.

In a sense, this paper is a guide to the interpretation of observational data, particularly as warnings as to how observations can be misinterpreted and misused. It is a review only in the sense of using the past to warn the future. To focus only on cataloging past observations would be to reduce the paper's value to the future.

The logical fallacies committed are not unique to the science of DBS in particular or to science in general. Rather, many have been known and extensively and rigorously studied for millennia. The understanding of these fallacies is embodied in the philosophical disciplines of epistemology and logic. Indeed, in many ways logic has the same rigor as mathematics, and just as no scientists would reject the possible contributions of mathematics, similarly scientists should not reject out of hand the contributions of epistemology and logic. Just as mathematical errors render inferences suspect so do logical errors. In the long history of epistemology and logic certain general forms of arguments have been found faulty, such as, the expression *if* ***a*** *implies* ***c*** *and* ***b*** *implies* ***c*** *therefore* ***a*** *implies* ***b*** is logically fallacious (the Fallacy of Pseudo-transitivity) regardless of what specific statements are substituted for ***a***, ***b***, and ***c***. Thus, one approach in this discussion will be to re-couch many of the claims related to the mechanisms of action of DBS into their logical equivalents of well known forms by which to demonstrate their being logical fallacies.

The paradox is that logical fallacies are integral and necessary for much of scientific progress yet at the same time, they have been the source of many misconceptions that has limited DBS research, as will be shown. For example, the Fallacy of Pseudo-transitivity was seen in equating the similar clinical benefits between pallidotomy and pallidal DBS with similarity of mechanisms, specifically that DBS inhibited neuronal activity or cause a “functional lesion.” This presupposition was a set back. However, Mary Walker used to the Fallacy of Pseudo-transitivity to equate weakness of curare poisoning with the weakness of Myasthenia gravis. She went on to hypothesize the anti-cholinesterase medications, which benefited patients with curare poisoning, would help patients with Myasthenia gravis, and fortunately, it did. The difference in these circumstances was the in the case of Myasthenia gravis, the Fallacy of Pseudo-transitivity was used to generate a hypothesis, and not to support a hypothesis and particularly to discount alternatives, as in the case in DBS research.

Faulty presuppositions increase the risk that inherently false hypotheses are subjected to the scientific hypothetico-deductive method and what is taken as evidence, though, they are false. Some scientists may object, saying that the means of testing and rules of evidence are self-evident and they naturally engender universal acquiescence. But this is judging science by the ideal it hopes to attain and not the actual history of science, as will be demonstrated in the history of the science of DBS mechanisms.

Thus far, the therapeutic mechanisms of DBS have escaped ready and complete explanation despite the over 30 years since I. Cooper and colleagues first described DBS for movement disorders as is currently implemented for movement disorders (Cooper et al., 1980), although, direct electrical stimulation of the living human brain goes back at least to the late 1800's (Bartholow, 1874). This speaks loudly to the possibility that the wrong questions are being asked. That the wrong questions are being asked suggests that the presumptions inherent in or leading to the questions being asked are wrong.

## Resurgence of interest in DBS in the 1990's

The first actual published descriptions of DBS, as currently practiced, were published by Dieckmann (1979) for psychiatric disorders and by Cooper et al. (1980) for movement disorders. However, to the current generation, it appears that DBS was discovered in the late 1980's with the publications by Benabid et al. (1987) evidenced by the many references to Benabid as the discoverer of DBS (Williams, 2012). Rather, the important work by Benabid and colleagues lead to a resurgence in interest of DBS but primarily as an extension of renewed interest in ablative surgical therapies in the 1980's. DBS did not reach its current level of enthusiasm until the early 1990's (Hariz et al., 2010). The enthusiasm for DBS followed on the resurgence of interest in the mid 1980's of pallidotomies for the treatment of pharmacological refractory Parkinson's disease (PD). Because DBS did not require purposeful destruction of brain tissue and its potential for adjustments over time, DBS came to be seen as clinically preferable to pallidotomy.

So if the initial reason for excitement about DBS was derivative of a resurgence of excitement in pallidotomy, the question becomes why the resurgence of pallidotomy in the 1980's when it had been performed in essentially the same manner since at least the 1950's? It was not due to advances in surgical technique or the realization that there would be patients who have no alternatives. Indeed, leading functional and stereotactic neurosurgeons were using the same basic approach, such as ventriculograms, for targeting in the 1990's that were used in the 1960's (Hariz et al., 2010). Similarly it is not the case that suddenly in the late 1980's, alternative treatments, such as levodopa, started to fail at increasing rates. The long term complications and limitations of levodopa therapy were recognized in the 1970's.

The argument can be made that it was the evolution of notions about the pathophysiology of the basal ganglia in PD that shifted the intellectual environment for the natural selection of the idea of pallidotomy as an interesting therapy. The evolution and popularization of the GPi Rate theory provided an apparent cogent rationale as to why pallidotomy should work. This consilience between neurosurgery, neurology, and theories of pathophysiology is seen in the essay by Goetz et al. (1993). The targeting of the subthalamic nucleus (STN), initially for surgical ablation and later for DBS, was a direct result of the GPi Rate theory, which posited over-activity of the STN contributing to the pathological over-activity of the GPi. It turns out that these concepts are incorrect but those issues have been addressed elsewhere (Montgomery, 2007). Thus, the historical fact of contemporaneous development of the GPi Rate theory and the resurgence of interest in pallidotomy is likely to be more than coincidence and it is reasonable to suggest a causal connection.

If the inference above is true, the fact that a theory could make such a difference in providing medical care is striking. For many reasons, the GPi Rate theory captured the imagination of clinicians and scientists alike and was protected by the well-known phenomenon of Confirmation Bias, favoring observations supportive of pre-conceptions. The Confirmation Bias raised the bar for demonstrations of evidence to the contrary or to find support to explore alternatives. Studies in 1986 (Montgomery et al., 1986) but not replicated until 2009 (Wang et al., 2009) demonstrated parkinsonism in non-human primates in the absence of changes in neuronal activities predicted by the GPi Rate theory. Indeed, the GPi Rate theory seems to have withstood a very large amount of contrary data, for example, that pallidotomies do not produce involuntary movements, see review by Obeso et al. (2000).

What was so powerful about the GPi Rate theory that so many scientists, physicians, and surgeons were swayed? First it was a narrative that had strong intuitive appeal which human nature finds most irresistible, even in the face of contrary evidence (Johnson-Laird, 2006). This psychological predisposition to over-attribute explanatory power to intuitively appealing theories has been carefully documented in experimental psychological studies (Johnson-Laird, 2006) and scientists studying basal ganglia pathophysiology and DBS mechanisms are not somehow uniquely immune. Thus, it is possible that the over-attribution of explanatory power to the GPi Rate theory established the theory as a presupposition taken as fact. In the opinion of this author, the presupposition of the basic tenants of the GPi Rate theory have been to the detriment of theories of basal ganglia pathophysiology and subsequently, to theories regarding the mechanisms of action of DBS.

## Logical fallacies in DBS research

It is a matter of historical fact that one of the first hypotheses as to the therapeutic mechanism of action of DBS was that high frequency stimulation produced inhibition or suppression of neuronal activities in the stimulated target (Benazzouz et al., 1995). As there were as yet no direct observations of neuronal activities during stimulation, the claim was based on two factors. First was the similarity of clinical effects between destructive lesions and DBS. Thalamotomy and pallidotomy produced the same clinical changes as did DBS of the ventrolateral thalamus (VL) and GPi, respectively. Second, the notion of high frequency DBS inhibiting or suppressing neuronal activities in the GPi and in the STN resonated with the GPi Rate theory. It is important to note that the hypothesis that high frequency DBS inhibits or suppresses the neurons in the target stimulated was not just a hypothesis viewed as tentative as any other. Rather, this hypothesis had the epistemic status of a fact as evidenced by the difficulty several investigators had trying to offer alternative but equally support hypotheses.

To be sure, there have been a number of studies that demonstrate reduced neuronal activities in structures subject to high frequency DBS (Boraud et al., 1996; Dostrovsky et al., 2000; Beurrier et al., 2001; Kiss et al., 2002) as well as studies demonstrating the contrary (Dostrovsky et al., 1999; Carlson et al., 2010). However, science is not a “majority vote” and one approach, barrowed from philosophy, for adjudicating between contrary evidence is discussed latter.

The inference from the similarity of clinical responses between thalamotomy and pallidotomy to VL DBS (which will be used as shorthand for DBS in the vicinity of the VL) and GPi DBS (which will be used as shorthand for DBS in the vicinity of the GPi), respectively, can be reconstructed as the logical argument form if ***a*** *implies* ***c*** *and* ***b*** *implies* ***c*** *then* ***a*** *implies* ***b***. In this case, ***a*** refers to GPi DBS, ***b*** refers to pallidotomy and ***c*** refers to improvement in the symptoms of PD. The fallacy of this logical form described is called the Fallacy of Pseudo-transitivity (Goodwin and Johnson-Laird, 2008). The falsity is demonstrated when one substitutes for ***a*** “stroke,” ***b*** “curare,” and ***c*** “weakness.” Certainly stroke implies weakness (***a*** *implies* ***c***) and curare implies weakness (***b*** *implies* ***c***) but one would not conclude that stroke implies curare (***a*** *implies* ***b***). Consequently, the Fallacy of Pseudo-transitivity is not a method of scientific validation. It is not evidence in support of the hypothesis that high frequency DBS inhibits the target structure.

The Fallacy of Pseudo-transitivity does have positive value in the generation of hypotheses. For example, Mary Walker suggested the fallacy by hypothesizing that just as some mechanism of curare poisoning implies weakness and some mechanism of myasthenia gravis implies weakness, then curare poisoning implies myasthenia gravis. As anti-cholinesterase inhibitors improve curare perhaps they might improve myasthenia gravis, and to patients' great relief, the anti-cholinesterases were found to do so. In this case, the Fallacy of Pseudo-transitivity was beneficial, but note that it was used for hypothesis generation and not as scientific validation as many have used for the notion that high frequency DBS inhibits.

There is evidence from a number of studies that DBS activates the output from the stimulated targets. For example, STN DBS (which will be used as shorthand for DBS in the vicinity of the STN) drives activity in GPi as evidenced by neuronal recordings in humans (Reese et al., 2011) and non-human primates and which, by the way, does not worsen or produce parkinsonism (Montgomery and Gale, 2008). STN DBS increases drive of the GPi as measured by cyclic guanosine monophosphate (cGMP) levels in micro-dialysis studies in humans (Stefani et al., 2005, 2006). There is evidence from animal studies and non-human studies that high frequency DBS of the GPi does not inhibit GPi at least as measured by the neuronal responses of neurons that receive GPi output such as the VL (Anderson et al., 2003; Montgomery, 2006).

To be sure, there have been a number of neuronal recording studies in targets being stimulated (Dostrovsky et al., 2000) and some such as the GPi show a reduction in GPi neuronal activities (Dostrovsky et al., 2000). Consequently, this is some dissonance between observations of reduced neuronal activity within the GPi while the GPi is being stimulated and inhibition of the VL with GPi DBS demonstrating driving of GPi output. This conundrum apparently has been resolved by the computational modeling of neurons in response to DBS demonstrating reduction in action potentials in the soma and dendrites while action potential generation at the axon hillock or first inter-node (McIntyre et al., 2004). The modeling recapitulated early demonstrations of similar phenomena in biological preparations (Coombs et al., 1957; Llinas and Terzuolo, 1964; Stuart and Hausser, 1994; Gulledge and Stuart, 2003; Dugladze et al., 2012).

The studies described above leave the question as to the primary mechanism of DBS; local inhibition of the GPi at the level of the soma and dendrites, which would be consistent with the GPi Rate theory, or increased GPi output with GPi DBS which would be inconsistent with the GPi Rate theory. To some degree, this debate has been resolved by the demonstration by R. Reese and colleagues of increased GPi activity with therapeutic STN DBS in a patient with PD (Reese et al., 2011). It is now clear that increased activity in the STN likely is not causal to Parkinsonism. Patients with unilateral STN DBS experience ipsilateral benefit despite increased drive of the contralateral STN with DBS of the ipsilateral STN. This increased drive is associated with both antidromic and orthodromic activation of the STN contralateral to the STN being stimulated (Walker et al., 2011).

Interestingly, a study of the response of neurons of the substantia nigra pars reticulata (SNpr) suggested decreased neuronal activity in the SNpr with STN stimulation in the rodent (Benazzouz et al., 1995). As the STN sends excitatory drive to the SNpr, the reduction of SNpr neuronal activity was taken as evidence of DBS inhibition of the SNpr. However, the problem was the neuronal activity in the SNpr was measured immediately following the stimulation train, as they were not able to remove the stimulus artifact. Studies of GPi neuronal activities with STN DBS in the non-human primate demonstrate increased neuronal activity that decreases below baseline immediately following the cessation of DBS (Montgomery, 2003). This suggests that what happens following DBS is not necessarily what is seen immediately following DBS and consequently, the findings by Benazzouz and colleagues described above and similar subsequent studies in humans (Lafreniere-Roula et al., 2010) have no bearing on what happens during DBS and the use of activity following DBS as evidence of what occurs during DBS is a logical fallacy.

Attribution of the changes in neuronal activity immediately following DBS to changes during DBS is a logical fallacy called the Category Error (Ryle, 2000). One wonders whether extrapolation of changes in metabolism that underlie fMRI and other neurometabolic imaging to inferences of underlying neuronal activity similarly risk the logical fallacy of the Category Error. Certainly, there is not a direct coupling of metabolism and neuronal activity (Logothetis, 2007). Indeed, some studies demonstrate decreased metabolism in the motor cortex in patients with PD during therapeutic DBS (Haslinger et al., 2005). Yet, STN DBS evoked potentials from electroencephalographic (EEG) recordings consistent with antidromic activation (Baker et al., 2002; Walker et al., 2012) argue for increased activity in the motor cortex. Increased motor cortex neuronal activity in non-human primates with STN DBS also has been demonstrated (Montgomery and Gale, 2008). The point here for scientists is that the conscientious scientist should exert caution, with a default position of skepticism, when inferring neuronal activity from neurometabolic imaging. Further, past claims DBS mechanisms drawn from neurometabolic imaging should be regarded as suspect, at least until proven otherwise.

Even a cursory survey will demonstrate that the vast majority of published studies on DBS mechanisms of action have been directed at the target stimulated. Even when neurons in other structures receiving monosynaptic inputs from the stimulated structure, the inferences are always in terms of the events going on within the stimulated structure. Papers are titled “Effects of STN DBS” as though the effects were entirely mediated by STN. It is rare to find a paper that describes the “Effects of DBS on structures in the vicinity of the STN.” It would be disingenuous to suggest that the latter was implied or implicit in the former.

It is now clear that DBS in the vicinity of the STN activates, rather than inhibits, many neurons and particularly the axons and axonal terminals, which have the lowest threshold for electrical stimulation. There is evidence that DBS in the vicinity of the STN activates axon terminals projecting from the motor and somatosensory cortex causing antidromic responses in these structures (Montgomery and Gale, 2008; Walker et al., 2012). STN DBS causes antidromic activation of the contralateral STN neurons (Walker et al., 2011). In addition, monosynaptic and polysynaptic activations percolate throughout the basal ganglia-thalamic-cortical system (Montgomery and Gale, 2008). Indeed, Vitek and colleagues demonstrate the DBS in the vicinity of the STN modulates neuronal activity in the cerebellar receiving areas of VL (Xu et al., 2008).

So the question arises, given the observations described above, how can anyone make any claims as to STN function as a consequence of DBS in the vicinity of the STN? The matter is no less problematic for DBS in the vicinity of the GPi, which also generates antidromic activation of VL neurons, which also is likely to generate orthodromic action potentials in axons of VL neurons projecting to the cortex and other areas (Montgomery, 2006). Yet a cursory survey of the published literature will demonstrate no paucity of such claims even without so much as a caveat.

It is striking how little concern there appears to be for the possibility of the DBS effects having nothing to do with the functions of the stimulated target. In fact, the benefit of STN DBS may have nothing to do with the STN. Studies in the rodent model of parkinsonism demonstrate that increases or decreases in STN neuronal activities have no effect on the parkinsonism (Gradinaru et al., 2009).

If the DBS effect is related to stimulation-induced neuronal changes percolated throughout the basal ganglia-thalamic-cortical system (systems effect), than attributing the functions of the system to a single structure is an example of a Mereological Fallacy, which is attributing to a part the function of the whole, on which Aristotle wrote. Consider the situation where there are five workers involved in each and every process of the operations of a machine. One could interfere with the actions of any one worker and the machine will fail. The fallacy is disrupting the activities of the first worker and when the machine fails, attributing the machine functions solely to the first worker. In terms of DBS science, the error is in studying one structure to the exclusion of others and then attributing the therapeutic mechanisms to the single structure studied. Even a cursory survey of the literature will demonstrate that the overwhelming majority of studies have been confined to the study of a single structure.

The Mereological Fallacy can be mitigated by systematic study of the many components within the relevant system, in this case the basal ganglia-thalamic-cortical system. One could argue that neurometabolic imaging is one such approach; however, to date, these methods lack the spatial and temporal resolution necessary to address the relevant time scale of the underlying dynamics. Alternative approaches could include the same manipulations within various components separately with outcome measures that assess the same physiological domain. For example, it is now clear that DBS of nearly all the components of the basal ganglia-thalamic-cortical system improve bradykinesia PD (Montgomery et al., 2011). However, there may be many forms of or mechanisms underlying bradykinesia, and thus, the phenomena of bradykinesia would have to be dissected. Demonstration that the same fundamental mechanisms are affected regardless of the site stimulated would argue for a “systems” effect rather than a “part” effect.

The Mereological fallacy is compounded by the Fallacy of Limited Alternatives. This fallacy is of the form ***a*** *and* ***b*** *are possible explanations of* ***c***, ***a*** *has been disproved, then* ***b*** *must be the explanation of* ***c***. Clearly, there may be any number of other explanations and ***b*** may very well be wrong as an explanation. However, just eliminating ***a*** seems to convey validity on ***b*** which is fallacious. Thus, demonstrating an effect of DBS on the neuronal activities of the structure stimulated seems to be enough to convince most scientists that the local effects somehow are necessary and sufficient. It might be sufficient because the local structure is part of the system but it is not necessary. Again, even a cursory review of the literature would demonstrate a paucity of publications where all the reasonable alternative hypotheses were discussed.

Another fallacy often committed in the science of DBS is the Fallacy of Confirming the Consequence. This fallacy often is combined with Conformation Bias. The Fallacy of Confirming the Consequence is of the form *if* ***a*** *implies* ***b*** *is true and* ***b*** *is true than* ***a*** *is true*. This form is false because ***b*** could be true for any number of reasons other than ***a***. One example would be the argument that if the GPi is overactive in PD then pallidotomy should improve PD. The observation is true that pallidotomy improves PD, which then is taken as evidence for the validity of the theory that overactivity of the GPi caused Parkinsonism, which is false. Note that the Fallacy of Confirming the Consequence sets up the Fallacy of Pseudo-transitivity that was seen as validating the equivalence between pallidotomy and pallidal DBS as described above.

If the consequences or observations are to be taken as proof of the hypothesis in the fallacy described above, then one can readily appreciate the dangers of being selective as to which consequent observation is taken as evidence. There is the human tendency to select those observations that are consistent with the hypothesis, known as Confirmation Bias and disregard, actively or passively, any contrary observations. Thus, the Confirmation Bias results in selecting a predicted observation (***b*** in the logical form above) that already presupposes the hypothesis (***a*** in the logical form). For example, the GPi Rate theory predicts improved PD with pallidotomy and consequently, the observation of improved PD with pallidotmy was taken as evidence of the GPi Rate theory. However, the GPi Rate theory also predicts that pallidotomy would worsen involuntary movements. As pallidotomy does not worsen involuntary movements, the GPi Rate theory must be wrong. Yet, Confirmation Bias lead to neglect of the contrary observation that pallidotomy did not worsen involuntary movements as evidenced by the persistence of the GPi Rate theory even after the contrary evidence was made clear, see review by Obeso et al. (2000). There is nothing in the observation of pallidotomy improving PD that trumps the observation that palidotomy does not worsen involuntary movements.

There are several prominent examples of passive Confirmation Bias in the failure to address certain observations that are clearly known but rarely discussed as they apply to DBS science. An important fact often overlooked in explanations of DBS actions is the short-term latencies to therapeutic effectiveness (as distinguished from long-term changes thought related to induced plasticity). For example, tremor responds to DBS within seconds, bradykineisa in PD within a few more but gait and balance may take tens of minutes. Such variability in latencies to effects is not amendable to explanation by simple depletion of neurotransmitters or accumulation of adenosine (Bekar et al., 2008) or any other neurohumoral type explanation.

An incredible conceptual insight generally not appreciated, is that DBS has or will change the nature of dynamics in neural systems so as to cause a sea change, as it will introduce true realistic dynamics of the basal ganglia-thalamic-cortical system. This will change appreciation for and understanding of dynamics, which is the change in systems or states over time courses on the order of milliseconds (ms) in distinction to current concepts of physiology and pathophysiology, which are static (Montgomery, 2004, 2007, 2010).

The importance of the dynamics on the millisecond time scale is clear when one considers that, for at least some patients with PD, 150 pulses per second (pps) DBS of the STN relieves the disability of PD while 100 pps DBS is ineffective (unpublished observations). The only difference is that the inter-stimulus spike interval is approximately 3 ms, that is 3/1000th of a second, shorter in the effective compared to ineffective DBS. There is no generally accepted theory that begins to account for why a 3 ms difference makes a difference.

The Fallacy of Confirming the Consequence is particularly problematic as it is the essence of the Scientific Method where hypotheses are tested and then modified if the experiments are inconsistent. Often unappreciated, the Scientific Method informs what to do when the consequences of the hypothesis are not confirmed, that is modify the hypothesis. However, unappreciated is that failure to disprove the hypothesis is not validation of the hypothesis (Popper, 2002). Therefore, an interesting paradox results when considering evidence for (consistent with prediction) or against (inconsistent with prediction). As supporting evidence does not demonstrate the validity of the hypothesis, then the hypothesis should be rejected solely on the basis of the contrary evidence, at least according to the Fallacy of Confirming the Consequence. Yet, that rarely is the case (Johnson-Laird, 2006).

The resolution of the paradox often is “majority vote” when the contrary evidence is not ignored outright. This has the perverse consequence that a flawed experiment replicated a thousand times trumps a valid experiment done once. One approach to addressing this paradox lies in attributing an observation to a causal or epiphenomenal relationship. However, this is fraught with difficulties. Another approach would be to barrow a philosophical approach by asking whether a hypothesized mechanism is a necessary or sufficient cause. A necessary cause is one that the effect (observation) always co-exists with the cause (the presumed mechanism). Thus, it takes only a single instance where there is not a co-existence to refute that the mechanism is necessary.

A condition may not be necessary but it may be sufficient. Now the expectation is not that the observation always be present with the condition, unlike the condition for necessity described above. However, the notion of sufficient cause does require that each time the presumed cause is present, then the observations likewise must be present.

Applying these considerations to DBS, there is the hypothesis that PD is related to increased oscillatory activity in the beta frequency range and that DBS reduces this oscillatory activity. However, it takes only a single example of a patient with PD lacking increase beta frequency oscillation (assuming no methodological error) to disprove increased beta frequency oscillation as a necessary cause, and thus, that DBS must act by reducing beta oscillations. Yet, review of the literature rarely described the presence or absence of beta oscillations in individual subjects and hence, evidence that increased beta oscillations is a necessary cause (Weinberger et al., 2009).

To demonstrate that increased beta oscillations cannot be a sufficient condition, it is only necessary to demonstrate that Parkinsonism can be absent when there are increased beta oscillations. If one assumes that DBS in the beta frequencies can drive synchronized activity in basal ganglia in the beta frequency, then the failure to worsen Parkinsonism would be evidence that increased beta oscillations is not a sufficient cause of Parkinsonism (Tsang et al., 2012). If increased oscillations in the beta frequency are neither a necessary or sufficient cause of parkinsonism, then it is epiphenomenal and reduction of beta frequency oscillation cannot be taken, prima facie, as a therapeutic mechanism of DBS.

## A call to re-examine assumptions and presuppositions

DBS truly is a revolutionary change in the treatment of neurological and psychiatric disorders. But its full potential is more likely to be realized if there is a clear understanding of its mechanisms of action. To date, hypotheses as to the mechanisms of action have been derivative from prevailing implicit presuppositions and explicit theories of pathophysiology, most of which are incorrect (Montgomery, 2007; Montgomery and Gale, 2008). An example is the presumption that high frequency DBS reduces neuronal activity in the stimulated target because Parkinsonism is due to overactivity of the STN and GPi. As discussed above, a therapeutic effect of DBS is reduction in beta oscillations because increased beta oscillations cause Parkinsonism.

These theories of pathophysiology, such as, the GPi Rate theory or the Beta Oscillation theory, constitute a risk for Confirmation Bias that has a high probability of generating false claims to knowledge. The risks of Confirmation Bias are greatly magnified by the use of logical fallacies because valid logic, which serves as a check on the evidence, is not used. To be sure, use of logical fallacies may be central to the process of hypothesis generation, as in the case of Mary Walker and myasthenia gravis as described above. However, when these fallacies are used as evidence, particularly against competing hypotheses, scientific progress can be impeded.

The clinical benefits of DBS are even more remarkable when on considers just how “dumb” current DBS is. Current systems are either “on” at some fixed frequency or “off.” It is indeed fortunate that the brain is much smarter as it only needs a bit of help from the dumb DBS. However, to achieve the full potential of DBS will require a entirely new conception of brain physiology. Though appreciated by L. Galvani (1737–1798), the notion that the nervous system is basically an electrical device seems to have been forgotten. The neurotransmitters, the basis for current neurohumoral conceptions of the brain (Arikha, 2007), are merely the messengers not the message and it is the abnormal messages that are causal to the dysfunction of most neurological and psychiatric disorders. The current emphasis to explain brain function and dysfunction in terms of neurotransmitters is like confining the discussion of computer functions to a discussion of the electrons alone.

Most physicians are comfortable prescribing medications that mimic neurotransmitters, and biologicals (therapeutic agents derived from living cells such as vaccines and tissue transplants) while they still view DBS with apprehension bordering on suspicion (personal observation). This may explain the fascination of gene therapy to change the neurotransmitters in the STN from glycine to GABA but results to date have not been nearly as effective as DBS (Kaplitt et al., 2007) and yet require as many or more penetrations of the brain than DBS making such gene therapy more risky than DBS. This bias will only change when physicians are as comfortable at prescribing electricity for the brain as they are at prescribing pills. For this to happen, the scientists and engineers will have to provide a cogent understanding of how DBS works which is not likely to happen if scientists and engineers cannot see the flaws of current theories and approaches.

### Conflict of interest statement

The author declares that the research was conducted in the absence of any commercial or financial relationships that could be construed as a potential conflict of interest.
